# Detection of *SS18-SSX1/2* fusion transcripts in circulating tumor cells of patients with synovial sarcoma

**DOI:** 10.1186/s13000-019-0800-x

**Published:** 2019-03-14

**Authors:** Joanna Przybyl, Matt van de Rijn, Piotr Rutkowski

**Affiliations:** 1 Department of Pathology, Stanford University School of Medicine, Stanford, CA USA; 2 Department of Soft Tissue/Bone Sarcoma and Melanoma, Maria Sklodowska-Curie Institute - Oncology Center, Warsaw, Poland

**Keywords:** Liquid biopsy, Circulating tumor cells (CTCs), Nested RT-PCR, SS18-SSX fusion transcript, Synovial sarcoma

## Abstract

A recent study on 15 patients with synovial sarcoma demonstrated very low prevalence of tumor-specific fusion transcripts in peripheral blood specimens. Our results in an independent cohort of 38 patients with synovial sarcoma support these findings. Synovial sarcoma patients could greatly benefit from a non-invasive monitoring of tumor burden by liquid biopsies. However, given the low detection rate of *SS18-SSX1/2* in circulation, we conclude that alternative markers other than the tumor-type specific fusion transcripts should be considered.

## Introduction

We have read with great interest the study on release of circulating tumor cells and cell-free nucleic acids in synovial sarcoma by Mihály et al. published in Diagnostic Pathology [1]. The authors reported the presence of *SS18-SSX2* fusion transcript in circulation of 1 of 15 patients (6.7%), which suggests that the presence of tumor-derived cell-free RNA or circulating tumors cells (CTCs) is a rare event in patients with synovial sarcoma. We performed a study in an independent cohort of 38 patients with synovial sarcoma and our results support the findings of Mihály et al. [1].

## Patients and methods

We analyzed 38 blood specimens collected between 2008 and 2011 from 28 females and 10 males with the median age of 35 years at diagnosis (range: 23–62 years). The size of primary tumor ranged from 1.5 to 15 cm. Approximately 9 ml (range: 5–14.5 ml) of peripheral blood was collected into EDTA tubes before the diagnostic biopsy and before the initiation of treatment. Blood cells were separated from plasma by centrifugation (3000 rpm, 10 min, at room temperature) within 2 h of the blood draw and the bottom fraction containing erythrocytes, leukocytes, platelets, and CTCs was used to extract total RNA with TRI Reagent BD (Sigma). We employed nested RT-PCR assay that allows for detection of one CTC in 1 ml of whole blood (estimated based on the serial dilutions of HS-SY-II cells carrying *SS18-SSX1* fusion in the whole blood of a healthy donor). One microgram of total RNA was reverse-transcribed with oligo(dT)_12–18_ primers and random hexamers using SuperScript II Reverse Transcriptase (Thermo Fisher Scientific). cDNA quality was assessed by PCR using *GAPDH* (glyceraldehyde-3-phosphate dehydrogenase) primers as described previously [2]. SS18-*SSX1/2* fusion junctions were detected using AmpliTaq Gold DNA Polymerase (Thermo Fisher Scientific) using primers described previously by Panagopoulos et al. [3]. The first round of amplification was performed with 2 μl of cDNA and the second round of amplification was performed using 5 μl of the first round PCR product. Both rounds of amplifications were performed in 50 μl volume. cDNA from 1273/99 cell line with *SS18-SSX2* fusion and frozen tumor specimen with validated *SS18-SSX1* fusion were used as positive controls. Purified PCR products were analyzed using the 3130xl Genetic Analyzer (Life Technologies/ Thermo Fisher Scientific). Despite certain differences in methodology employed by Mihály et al. and in our study (e.g., the use of heparinized blood collection tubes and enrollment of patients after treatment in the study of Mihály et al.), we demonstrated a comparable limit of detection of our protocols (1 CTC in 1 ml of peripheral blood) and a similar sensitivity of CTC detection in blood samples from patients with synovial sarcoma.

## Results

Nested RT-PCR demonstrated a baseline sensitivity of fusion transcript detection of 5.3% (2/38 patients) with a specificity of 100% (determined using blood samples obtained from the 13 healthy donors and 5 donors with rheumatoid arthritis). Both patients with *SS18-SSX1* fusion transcript detected in peripheral blood had the *SS18* rearrangement confirmed by FISH in their tumors. These patients were male with synovial sarcoma located in lower extremity: a 54-year-old patient with 5.5 cm tumor in foot and a 30-year-old patient with a 9.5 cm tumor in calf. Both patients had localized disease at the time of blood collection.

## Discussion

Patients with synovial sarcoma could greatly benefit from non-invasive assays for improved evaluation of response to treatment or early detection of disease recurrence. Our results, together with the report of Mihály et al., show that the liquid biopsy approach based on detection of *SS18-SSX1/2* fusion transcript alone is not sensitive enough in patients with synovial sarcoma. We have previously demonstrated a similar low sensitivity of CTC detection based on the analysis of *EWS-FLI1* and *EWS-ERG* fusion transcripts in patients with Ewing sarcoma [2]. In that study we detected CTCs only in 9% (3/34) of patients. Given this low rate of CTC detection in translocation-related sarcomas, we propose that other markers in peripheral blood, such as circulating tumor DNA (ctDNA), may be more clinically useful for an improved prognostication, molecular profiling, and surveillance of sarcoma patients. We have recently demonstrated that simultaneous detection of copy number changes and point mutations in ctDNA increased the number of molecular markers that can be monitored in plasma and may be clinically useful in leiomyosarcoma [4]. Synovial sarcomas also demonstrate certain genomic aberrations that could potentially be detectable in plasma DNA. For example, we previously reported that a subset of synovial sarcomas show extensive DNA copy number alterations that could be used as a marker in ctDNA in patients with an increased genomic instability in their tumors [5]. The Cancer Genome Atlas study has also demonstrated a unique DNA methylation profile of synovial sarcomas compared to the other types of sarcomas, and methylation patterns could also be profiled in ctDNA [6]. Thus, a strategy similar to the one that we applied in leiomyosarcoma for an integrated profiling of different classes of genomic aberrations in ctDNA could be considered also in patients with synovial sarcoma.

## Abbreviations

CTC: Circulating tumor cell
ctDNA: circulating tumor DNA
EDTA: Ethylenediaminetetraacetic acid
PCR: Polymerase chain reaction
rpm: Revolutions per minute
RT-PCR: Reverse transcription polymerase chain reaction
